# Chronic low back pain as a biopsychosocial disease: time to change our point of view

**DOI:** 10.1186/s44158-021-00010-x

**Published:** 2021-10-13

**Authors:** Arturo Cuomo, Marco Cascella, Alessandro Vittori, Franco Marinangeli

**Affiliations:** 1 Division of Anesthesia and Pain Medicine, Istituto Nazionale Tumori - IRCCS - Fondazione Pascale, Naples, Italy; 2 Department of Anesthesia and Critical Care, ARCO ROMA, Ospedale Pediatrico Bambino Gesù IRCCS, Piazza S. Onofrio 4, 00165 Rome, Italy; 3 Department of Anesthesiology, Intensive Care and Pain Treatment, University of L’Aquila, L’Aquila, Italy

Despite a plethora of available analgesic treatments, chronic low back pain (cLBP) remains the most frequent cause of disability worldwide [1, 2]. It is usually associated with multiple comorbid conditions also affecting the psychological component, such as depression, panic and anxiety disorders, and sleep disturbances [3]. Furthermore, taken together, this heterogeneous group of clinical conditions is accompanied by a significant socioeconomic burden in both high- and low-income countries. Consequently, despite the multiple guidelines on the subject [4], effective cLBP management has multiple gaps. These gaps are probably attributable to the paradox — on PubMed, a rough search with the words “low back pain” yielded 42,279 results — of poor knowledge about the pathophysiology, clinic, and epidemiology of the phenomenon and to health policies that, probably, require appropriate changes.

Although the precise pathophysiological bases of cLBP must be well elucidated, they encompass complex combinations of peripheral pathological mechanisms and different alterations of neural and brain structures [5]. Damages mostly due to degeneration processes of the intervertebral discs, facet joints, muscles, tendons, ligaments, fascia (lumbar muscle fascia), synovium, and joint capsules can produce nociceptive and/or neuropathic pain (e.g., through nerve compression). Nevertheless, mechanical compression and peripheral inflammatory factors trigger a cascade of neurobiochemical reactions. These alterations can finally induce peripheral and central phenomena of sensitization which are expressed as pain in the absence of noxious stimuli [6]. About central mechanisms, cortical, subcortical, and spinal cord areas are involved with changes in structural and functional plasticity as well as central pain modulation network abnormalities [7]. Since alterations can involve different brain areas, sensory, emotional, cognitive, and behavioral elements contribute, with a different weight, to define the features of chronic pain [8].

In the lack of an exhaustive understanding of chronic pain, several theories have been formulated over the years to explain the phenomenon. In 1999, in a fascinating paper titled “From the gate to the neuromatrix,” the psychologist Ronald Melzack proposed a model which led to a new notion of chronic pain [9]. Notably, in 1965, Melzack and Wall illustrated the famous “gate control theory” that firstly viewed pain as a mind-body perspective [10]. According to the concept of neuromatrix, multiple areas of the central nervous system work together to produce pain and, in turn, chronic pain does not originate from tissue damage, but from the brain itself [9]. In this view, cLBP is not considered a linear experience directly induced by sensory input, but rather it is conceived as a multidimensional process evoked by a compounded neural network [11]. In recent decades, other theories and research have been published, but the mosaic of the pathophysiology of cLBP is far from being completed.

Chronic pain goes beyond the boundaries of those who experience it. Increasing evidence suggests the major impact of cLBP on both the affected patient and his/her family and social environment [12]. Consequently, cLBP can be considered a “biopsychosocial issue” [11]. The biopsychosocial model is another paramount pain theory. Every experience of cLBP remains unique and peculiar for the single patient and evolves dynamically. In this line, the International Association for the Study of Pain (IASP) released, in 2019, the International Classification of Diseases 11th revision (ICD-11). In particular, the IASP revised the classification of chronic pain and offered conceptual instruments useful for the diagnosis and the individualized management of primary and secondary chronic pain in different settings [13]. Interestingly, in the ICD-11, the severity of pain is rated according to three distinct dimensions namely the intensity of pain, pain-related distress, and interference with daily living. The patient rates each dimension according to a 0–10 scale (0: absent; 1-3: mild; 4–6: moderate; 7–10: severe). Hence, the pain state is given by the sequence of a 3-digit code, which allows evaluating the severities of each dimension. This strategy could induce a positive trend reversal because much of the failure in managing cLBP is probably attributable to the lack of a holistic approach to the problem.

The clinical assessment of cLBP is crucial. Given the recognized multidimensional nature of pain, it is mandatory to evolve the strategy of pain management and its aims, from the mere relief of pain intensity to the restoring of functionality and psychological well-being [11, 14, 15]. In particular, the complexity of cLBP immediately leads to the need for a multidisciplinary assessment. In daily practice, different specialists contribute to the management of cLBP including general practitioners, orthopedists, pain therapists, physiatrists, and others. Each of these healthcare providers has different objectives and experiences. In the management of cLBP, it is fundamental to properly define the specific responsibilities of each involved specialist [14].

The most important aspect concerns the therapeutic pathway. Chronic pain should be managed through a combination of pharmacological and nonpharmacological methods keeping into consideration its intensity, pathophysiology, the complexity of symptoms, the presence of comorbidity, the social context, and the duration of disease illness. In 2019, we proposed a simple and intuitive model for pain relief, defined as “the analgesic trolley” [11]. In this model, several strategies can be applied to the treatment of pain, and therefore also to cLBP, according to the specific needs of each patient and the underlying pain mechanisms [16].

Proper treatments must be planned and started early. Although it is widely accepted that early treatment of cLBP is highly important in clinical practice, the delay in initiating adequate therapy represents a great issue [8]. It is often underestimated that proper pain treatment results in improved physical and psychological well-being, and also in a recovery of functionality, with restoring of the capability to perform daily activities, increased working ability, and diminished need for other medical therapies.

Regardless of the therapeutic strategy, pain treatment must focus on restoring functionality and physiological well-being. In harmony with the World Health Organization definition of health that is intended as a “state of complete physical, mental and social well-being and not merely the absence of disease or infirmity” and other new proposed definitions [17, 18], functionality can be viewed as the ability to ambulate, function cognitively, return to work, and complete activities of daily living including the absence of mood and sleep disturbances. This point of view has paramount implications for practice, policy, and health services since the management of functional impairment in patients with chronic pain is a collection of unmet needs concerning assessment, therapy, and rehabilitation processes.

Although applying these concepts in daily clinical practice is a great challenge, the right pathway seems to be drawn. For this purpose, Scientific Societies should implement suitable strategies to structure multiprofessional protocols and enhance the training of pain specialists and other professionals. Easy to apply guidelines and practical tools useful in different care settings are needed. Finally, the Scientific Societies must promote high-impact clinical research, involving all elements of the pain care network, in a capillary way.
